# Efficacy and Safety of Gemcitabine-Fluorouracil Combination Therapy in the Management of Advanced Pancreatic Cancer: A Meta-Analysis of Randomized Controlled Trials

**DOI:** 10.1371/journal.pone.0104346

**Published:** 2014-08-05

**Authors:** Qin Li, Han Yan, Wenting Liu, Hongchao Zhen, Yifan Yang, Bangwei Cao

**Affiliations:** 1 Department of Oncology, Beijing Friendship Hospital, Capital Medical University, Beijing, China; 2 Beijing Key Laboratory for Precancerous Lesion of Digestive Diseases, Beijing Friendship Hospital, Capital Medical University, Beijing, China; 3 Beijing Digestive Diseases Center, Beijing Friendship Hospital, Capital Medical University, Beijing, China; Thomas Jefferson University, United States of America

## Abstract

**Background:**

Gemcitabine (GEM) is the standard first-line chemotherapy that provides limited clinical benefits for patients with locally advanced/metastatic pancreatic adenocarcinoma (LA/MPC). However, the fluorouracil derivatives (CAP and S-1) show promising efficacy in these patients. This study compared the efficacy and safety of GEM with GEM plus fluorouracil drugs in the treatment of LA/MPC.

**Methods:**

Pubmed, EMBASE and Cochrane Library databases were searched for relevant randomized controlled trials published on or before January 2014. The Cochrane Collaboration's tool was used to assess the risk of bias in randomized trials. The primary end point was overall survival (OS); the secondary end points were one-year survival rate, objective response rate (ORR) and toxicity rates (TRs).

**Results:**

A total of 8 randomized controlled trials involving 2,126 patients were included in the systematic evaluation. The results showed that OS was significantly improved (HR 0.83, *P*<0.01; HR 0.87, *P* = 0.03; HR 0.80, *P* = 0.01; respectively) and ORR was significantly increased (OR 0.51, *P*<0.01; OR 0.66, *P* = 0.03; OR 0.35, *P*<0.01; respectively) in the GEM+5-FU/CAP/S-1, GEM+CAP and GEM+S-1 groups compared to the GEM alone group. In addition, the one-year survival rate was significantly increased (OR 0.78 *P* = 0.01; OR 0.47, *P* = 0.04; respectively) in the GEM+5-FU/CAP/S-1 and GEM+S-1 groups compared to the GEM alone group. The frequency of grade 3/4 TRs were higher in GEM+5-FU/CAP/S-1 group, the significant increase of grade 3/4 neutropenia, thrombocytopenia and diarrhea were observed.

**Conclusions:**

GEM combined with fluorouracil drugs significantly improved OS and increased one-year survival rate and ORR compared to GEM alone in LA/MPC patients. GEM combined with fluorouracil drugs may be considered as an acceptable alternative treatment for LA/MPC patients.

## Introduction

Pancreatic cancer is the eighth leading cause of cancer-related mortality worldwide. More than 80% of patients with pancreatic cancer have late-stage disease when diagnosed. Patients with locally advanced/metastatic pancreatic adenocarcinoma (LA/MPC) have missed the opportunity to be managed surgically [1]. Gemcitabine (GEM) is the standard first-line chemotherapy for patients with LA/MPC, offering a statistically longer survival compared to 5-fluorouracil (5-FU) [2], [3]. However, the prognosis of patients with LA/MPC remains poor. In order to achieve a better survival benefit for LA/MPC patients, GEM combined with cytotoxic drugs or molecular-targeted agents has been intensely investigated.

Many studies on GEM-based combination scheme have failed to demonstrate an improvement in overall survival (OS) [2], [4], [5]. Only a minority of combination therapies such as GEM plus erlotinib or GEM plus nab-paclitaxel showed a significant improvement of OS compared to GEM alone in LA/MPC patients [6], [7]. Three meta-analyses showed that GEM combination chemotherapy conferred a significant benefit in terms of OS or a modest improvement of one-year survival rate compared to GEM mono-therapy in LA/MPC patients. However, these combination therapies were associated with increased toxicity [8]–[10]. This combination therapy offers some viable options for the management of LA/MPC patients with good performance status.

The aforementioned meta-analyses included GEM combined with biologics or cytotoxic agents, however, our study focused on GEM combined with fluorouracil drugs compared to GEM alone. Fluorouracil drugs including 5-FU, Capecitabine (CAP) and S-1 have proven to be effective in LA/MPC treatment. Two randomized controlled trials (RCTs) reported that the median survival times (MST) were 4.2 months and 5.1 months respectively and one-year survival rates were 26% and 23% respectively for LA/MPC patients receiving protracted venous 5-FU infusion [11], [12]. Similar survival rates were obtained using protracted venous infusion of 5-FU and GEM, and this supported further exploration of the role of fluorouracil in LA/MPC patients. CAP is an oral fluorouracil pro-drug that has selective activity against tumor cells and it exerts sustained antitumor effects when transformed into 5-FU. Cartwright et al. reported that treatment with CAP resulted in a clinically significant benefit, with a MST of 6 months in LA/MPC patients [13]. This result together with its generally tolerable safety profile and the added advantage of oral administration provide the basis for further evaluating CAP in combination with GEM in this patient population. S-1 is a newly developed oral 5-FU derivative, which contains tegafur, gimeracil and oteracil potassium. Gimeracil enhances S-1 anti-tumor effect by preventing its degradation and oteracil potassium reduces digestive tract reactions by protecting the gastrointestinal mucosa. The efficacy of S-1 has already been demonstrated on a variety of solid tumors [14], [15]. A phase II trial of S-1 alone in MPC showed a response rate of 37.5% and a MST up to 9.2 months, which far exceeded the efficacy of GEM [16].

Because fluorouracil drugs have shown promising activity in LA/MPC patients, many RCTs have been designed to evaluate whether GEM combined with fluorouracil drugs is superior to GEM alone, but the conclusions are not consistent. Therefore, we undertook a systematic assessment of relevant RCTs in this study.

## Materials and Methods

### Literature search strategy

This meta-analysis was performed according to the Preferred Reporting Items for Systematic Reviews and Meta-analysis (PRISMA) criteria [17]. PubMed, EMBASE and the Central Registry of Controlled Trials of the Cochrane Library were searched for original articles written in English and published before January 31, 2014. Abstracts presented at the annual meeting of the American Society of Clinical Oncology and the European Cancer Conference were also searched. Prospective studies were allowed in this assessment to minimize the risk of selection or information bias. The initial search used the MeSH terms “Pancreatic neoplasm OR Pancreas neoplasm OR Pancreas Cancers OR Pancreatic Cancer OR Pancreatic Carcinoma” AND “Gemcitabine OR Gemzar” AND “Fluorouracil OR 5-Fluorouracil OR 5-FU; Capecitabine OR Xeloda; S-1 OR S1”.

### RCT selection and exclusion criteria

The inclusion criteria were as follows: (1) the trials were required to be prospective, properly randomized and well-designed, which we defined as matched for age, gender, tumor stage and performance status (PS) or Karnofsky performance status (KPS); (2) the subjects of the trials were patients with LA/MPC, and histologic or cytologic confirmation of pancreatic adenocarcinoma was required; (3) the patients received GEM monotherapy in the control arm, while patients received GEM combined with 5-FU/CAP/S-1 therapy in the experimental arm; (4) the primary end point was OS, secondary end points were one-year survival rate, objective response rate (ORR) and toxicity rates (TRs); (5) the original article had explicit survival information included as follow-up censored or explicit survival curves, and the follow-up rate was greater than 95%; and (6) whenever trials with overlapping patient populations were encountered, only the trial with the longest follow-up was included.

The exclusion criteria were: (1) trials that included patients with major comorbidities or second tumors were excluded; and (2) if a trial included adjuvant chemotherapy within six months or concomitant interventions such as radiotherapy that differed systematically between the investigated arms, it was excluded.

### Data collection and extraction

All identified abstracts were assessed independently by two investigators (Qin Li and Yi-fan Yang). If one investigator considered that an abstract was eligible, the full text of the article was retrieved and reviewed in detail by both investigators. Any discrepancy was resolved by an arbiter (Han Yan) or by contacting the authors of the original study. Different variables including authors' names, journal, year of publication, original country, sample size per arm, performance status, regimens, line of treatment, median age of patients, gender ratio, tumor stage and pre-specified outcomes of efficacy and safety were extracted and evaluated.

### Assessment of methodological quality

Following the Cochrane Handbook for Systematic Reviews of Interventions [18], the methodological quality of the included studies was independently assessed by two authors. Any disagreements were resolved by discussion. The corresponding author was the arbiter when no consensus could be achieved. We evaluated the risk of bias in the studies using the Review Manager software (RevMan Version 5.1; The Nordic Cochrane Center, The Cochrane Collaboration, Copenhagen, Denmark), which included the following key domains: random sequence generation (selection bias), allocation concealment (selection bias), blinding of participants and personnel (performance bias), blinding of outcome assessment (detection bias), incomplete outcome data (attrition bias), selective reporting (reporting bias) and other bias. The publication bias was assessed using funnel plots.

Within a trial, low risk of bias for all key domains indicated a low risk of bias, low or unclear risk of bias for all key domains indicated an unclear risk of bias, and high risk of bias for one or more key domains indicated a high risk of bias. Across trials, most information from trials at low risk of bias indicated a low risk of bias, most information from trials at low or unclear risk indicated an unclear risk of bias, and that the proportion of information from trials at high risk of bias was sufficient to affect the interpretation of results indicated a high risk of bias.

### Statistical analysis

The systematic assessment was performed using Review Manager Version 5.1.7 (http://ims.cochrane.org/revman). Heterogeneity between the trials was assessed to determine which model should be used. The Cochrane's *Q*-test was performed and *I^2^* statistics were obtained, with a predefined significance threshold of 0.05. A *P* value of more than 0.05 suggested that the studies were homogeneous, and the pooled estimation of hazard ratio (HR) and odds ratio (OR) for each study were calculated using the fixed effects model (FEM). A *P* value of less than 0.05 for the *Q*-test suggested that the studies were heterogeneous, and the random effects model (REM) was applied. HR and OR were the principal measurements of effect and were presented with 95% confidence interval (CI); a *P* value of less than 0.05 was considered statistically significant. All reported *P* values were from two-sided versions of the respective tests. If a trial provided only a Kaplan-Meier curve, the HR and 95% CI were estimated utilizing the Engauge Digitizer V4.1 screenshot tool and a formula proposed by Parmar [19], [20]. The potential presence of publication bias was evaluated visually by inspecting funnel plots and statistically using the Egger's test.

## Results

### Selection of the trials

**Figure 1:**
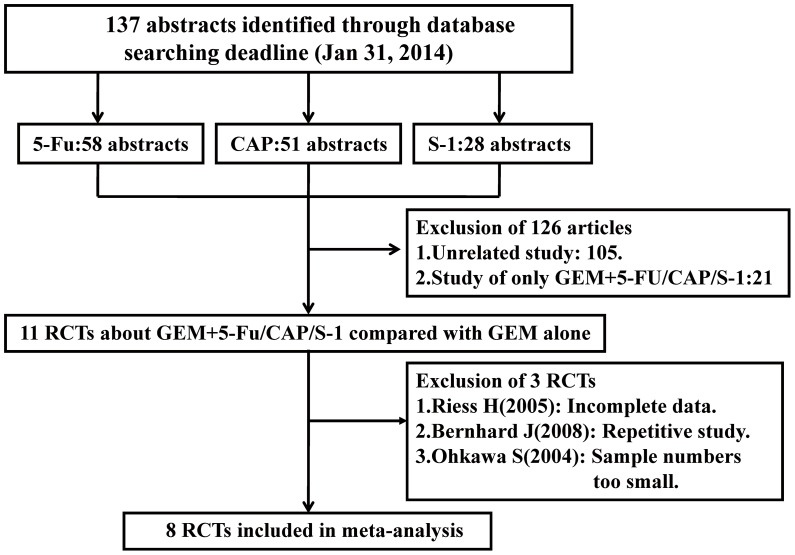
PRISMA flow diagram showing the exclusion and inclusion of trials in the meta-analysis.

The inclusion and exclusion of RCTs for this systematic assessment are shown in a flow chart (Figure 1). In accordance with our search strategy, 137 abstracts were screened. Primary screening led to the exclusion of 126 abstracts for the following reasons: 105 abstracts were unrelated studies and 21 abstracts were only single-arm studies about GEM combined with 5-FU or CAP or S-1. The remaining 11 articles were retrieved for more detailed evaluation. Of these, 3 articles were excluded due to incomplete data, repetitive study or small sample size. In the end, 8 RCTs were eligible for inclusion in this meta-analysis. The PRISMA checklist is shown in Checklist S1.

### The risk of bias in the included studies

**Figure 2:**
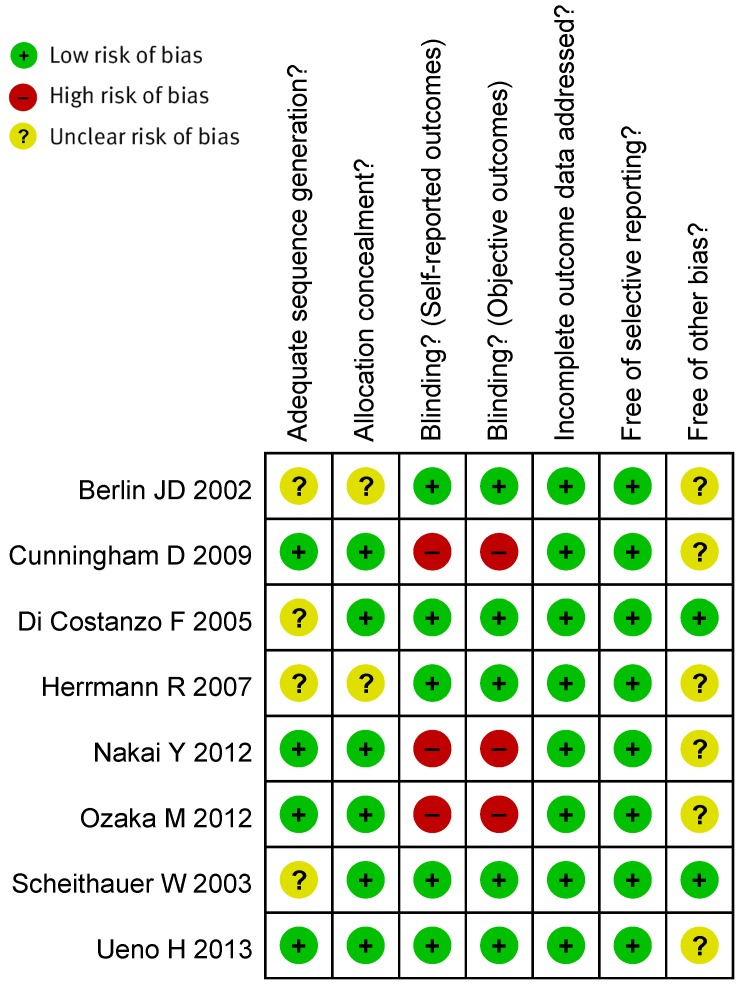
The risk of bias for the included studies.

Four RCTs were assessed to have an unclear risk of selection bias due to insufficient detail on random sequence generation or allocation concealment. Three RCTs were assessed to have a high risk of performance and detection bias due to open label in trial design. Six RCTs were assessed to have an unclear risk of other bias due to insufficient details, such as lacking an adequate description of patients' the uptake of the therapeutic drug monitoring recommendations by physicians (Figure 2).

### Main characteristics of RCTs included in the systematic assessment

The main characteristics of all eligible RCTs are listed in Table 1. Of the eight trials, four were randomized phase II trials and four were randomized phase III trials. A total of 2,126 patients were included in this assessment, of which 1,059 patients received GEM+5-FU/CAP/S-1 therapy and 1,067 patients received GEM alone therapy. In subgroup analysis, 416 patients received GEM+5-FU versus GEM alone therapy, 935 patients received GEM+CAP versus GEM alone therapy, and 775 patients received GEM+S-1 versus GEM alone therapy. The data on OS, ORR and TRs were extracted from eight trials and the data on one-year survival rates were extracted from seven trials.

**Table 1:** Characteristics of the eligible trials included in the systematic assessment.

Trial	Phase	Arms	Case (n)	Male (%)	Median age (y)	LA (%)/MPC (%)	Regimens
Berlin JD	III	GEM+5-FU	160	51.8	65.8	11/89	GEM 1000 mg/m^2^, then 5-FU 600 mg/m^2^ d1,8,15, q4w, IV.
2002 [21]	Multicenter	GEM alone	162	53.7	64.3	10/90	GEM 1000 mg/m^2^ d1,8,15, q4w, IV.
Scheithauer W	II	GEM+CAP	41	66.0	64.0	0/100	GEM 2200 mg/m^2^ d1, q2w, IV; CAP 2500 mg/m^2^ d1-7, q2w, PO.
2003 [22]	Multicenter	GEM alone	42	55.0	66.0	0/100	GEM 2200 mg/m^2^ d1, q2w, IV.
Di Costanzo F	II	GEM+5-FU	45	63.0	62.0	33/67	GEM 1000 mg/m^2^/w, 5-FU 200 mg/m^2^/d×6weeks followed by 1-week rest; then d1,8,15, q4w, IV.
2005 [23]	Multicenter	GEM alone	49	48.0	64.0	27/73	GEM 1000 mg/m^2^/w×7 weeks followed by 2-weeks rest, then d1,8,15, q4w, IV.
Herrmann R	III	GEM+CAP	160	54.0	Unknown	20/80	GEM 1000 mg/m^2^ d1,8, q3w, IV; CAP 650 mg/m^2^ twice daily d1-14, q3w, PO.
2007 [24]	Multicenter	GEM alone	159	53.0	Unknown	21/79	GEM 1000 mg/m^2^/w×7 weeks followed by 1-week rest, then d1,8,15, q4w, IV.
Cunningham D	III	GEM+CAP	267	60.0	62.0	30/70	GEM 1000 mg/m^2^ d1,8,15, q4w, IV; CAP 830 mg/m^2^ twice daily d1-21, q4w, PO.
2009 [25]	Multicenter	GEM alone	266	58.0	62.0	29/71	GEM 1000 mg/m^2^/w×7 weeks followed by 1-week rest, then d1,8,15, q4w, IV.
Nakai Y	II	GEM+S-1	53	79.2	63.0	28/72	GEM 1000 mg/m^2^ d1,15, q4w, IV; S-1 40 mg/m^2^ twice daily d1-14, q4w, PO.
2012 [26]	Multicenter	GEM alone	53	62.3	67.0	24/76	GEM 1000 mg/m^2^ d1,8,15, q4w, IV.
Ozaka M	II	GEM+S-1	58	60.3	Unknown	25/75	GEM 1000 mg/m^2^ d1,8, q3w, IV; S-1 40 mg/m^2^ twice daily d1-14, q3w, PO.
2012 [27]	Multicenter	GEM alone	59	59.3	Unknown	31/69	GEM 1000 mg/m^2^ d1,8,15, q4w, IV.
Ueno H	III	GEM+S-1	275	57.5	Unknown	25/75	GEM 1000 mg/m^2^ d1,8, IV; S-1 60/80/100 mg/m^2^ d1-14, q3w, PO.
2013 [28]	Multicenter	GEM alone	277	61.4	Unknown	24/76	GEM 1000 mg/m^2^ d1,8,15, q4w, IV.

GEM, gemcitabine; 5-FU, 5-fluorouracil; CAP, capecitabine; LA/MPC, locally advanced/metastatic pancreatic adenocarcinoma; OS, overall survival.

### Efficacy analysis

Four RCTs, including one GEM+CAP versus GEM trial and three GEM+S-1 versus GEM trials, provided complete data on OS [HR (95% CI)]. Four RCTs, including two GEM+5-FU versus GEM trials and two GEM+CAP versus GEM trials, provided only the OS and Kaplan-Meier curves. The Engauge Digitizer V4.1 screenshot tool and the formula proposed by Parmar et al were used to estimate the HR (95% CI).

**Figure 3:**
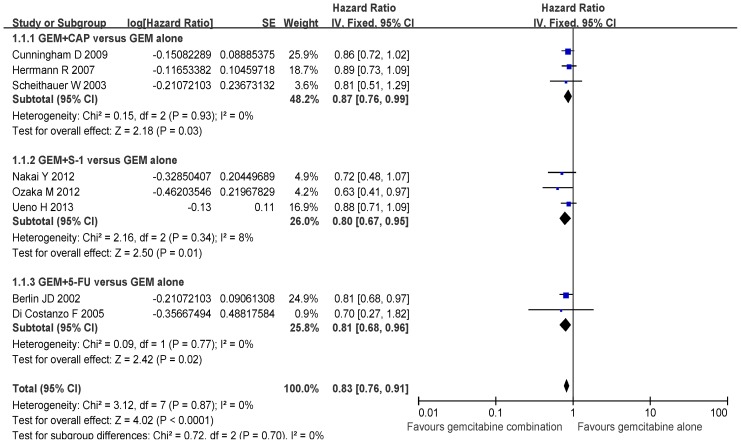
Comparison of overall survival between GEM combination therapy and GEM alone therapy.

There was no significant difference in the heterogeneity for OS between the GEM combination group and the GEM alone group (*P*>0.05), and therefore FEM was selected for this systemic assessment. The analysis indicated a significant improvement in OS when the GEM+5-FU/CAP/S-1, GEM+CAP, GEM+S-1, GEM+5-FU groups were compared to the GEM alone group (HR 0.83, 95% CI: 0.76–0.91, *P*<0.01; HR 0.87, 95% CI: 0.76–0.99, *P* = 0.03; HR 0.80, 95% CI: 0.67–0.95, *P* = 0.01; HR 0.81, 95% CI: 0.68–0.96, *P* = 0.02; respectively) (Figure 3).

**Figure 4:**
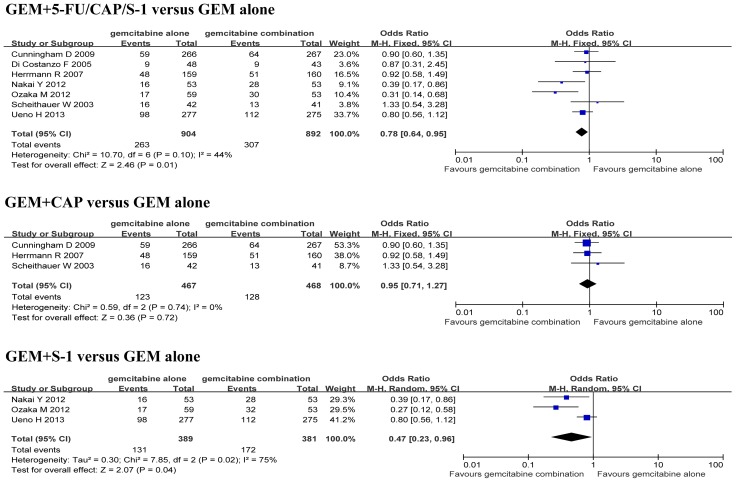
Comparison of one-year survival rate between GEM combination therapy and GEM alone therapy.

There was no significant difference in the heterogeneity for one-year survival rates between the GEM+5-FU/CAP/S-1, GEM+CAP groups and the GEM alone group (*P*>0.05), and therefore FEM was selected. However, there was a significant difference in the heterogeneity for one-year survival rates between the GEM+S-1 group and the GEM alone group (*P*<0.05), so REM was applied. The analysis indicated a significant increase in one-year survival rate when the GEM+5-FU/CAP/S-1 and GEM+S-1 groups were compared to the GEM alone group (OR 0.78, 95% CI: 0.64–0.95, *P* = 0.01; OR 0.47, 95% CI: 0.23–0.96, *P* = 0.04; respectively) (Figure 4). However, there was no significant difference in one-year survival rate when the GEM+CAP group was compared to the GEM alone group (OR 0.95, 95% CI: 0.71–1.27, *P* = 0.72) (Figure 4).

**Figure 5:**
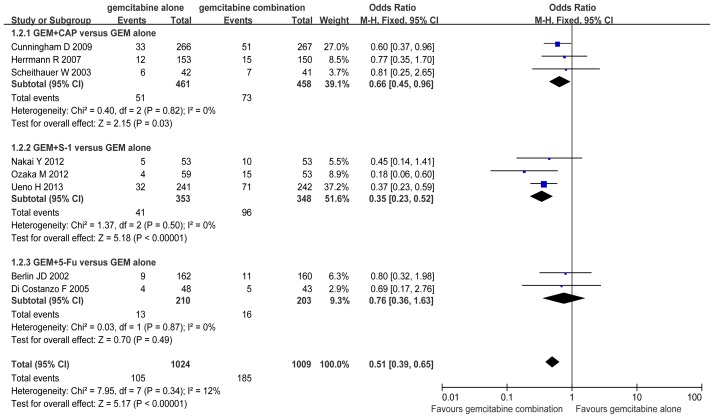
Comparison of objective response rate between GEM combination therapy and GEM alone therapy.

There was no significant difference in the heterogeneity for ORR between the GEM combination groups and the GEM alone group (*P*>0.05), and therefore FEM was applied. The analysis demonstrated a significant increase in ORR when the GEM+5-FU/CAP/S-1, GEM+CAP and GEM+S-1 groups were compared to the GEM alone group (OR 0.51, 95% CI: 0.39–0.65, *P*<0.01; OR 0.66, 95% CI: 0.45–0.96, *P* = 0.03; OR 0.35, 95% CI: 0.23–0.52, *P*<0.01; respectively) (Figure 5).

### Efficacy analysis of phase III trials

**Figure 6:**
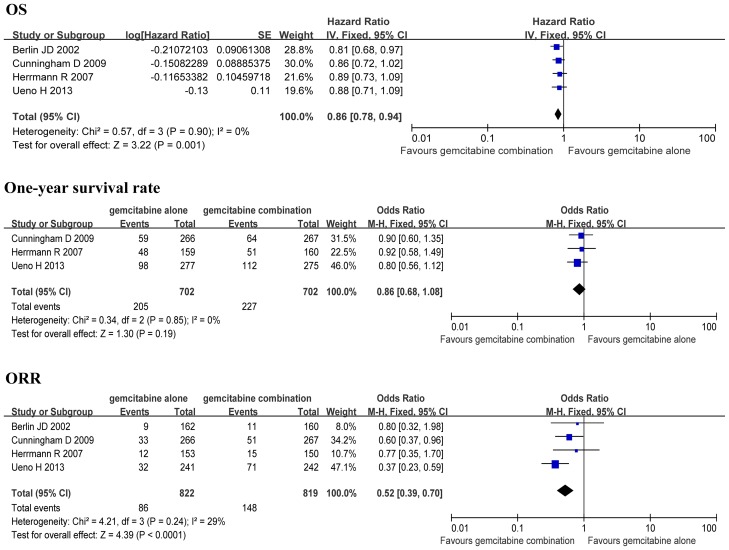
Efficacy sub-analysis of the phase III trials.

The efficacy of four phase III trials, including one GEM+5-FU versus GEM trial, two GEM+CAP versus GEM trials and one GEM+S-1 versus GEM trial, was analyzed. The analysis showed a significant improvement in OS (HR 0.86, 95% CI: 0.78–0.94, *P*<0.01) and a significant increase in ORR (OR 1.91, 95% CI: 1.43–2.54, *P*<0.01) when the GEM+5-FU/CAP/S-1 group was compared to the GEM alone group (Figure 6).

### Grade 3–4 toxicity analysis

**Figure 7:**
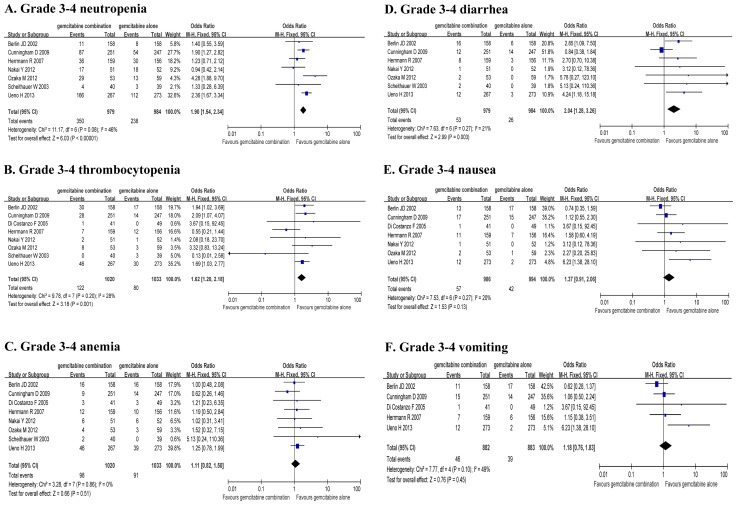
Comparison of Grade 3–4 toxicity rates between GEM combination therapy and GEM alone therapy.

Grade 3–4 hematologic adverse events, gastrointestinal reactions and other toxicities were extracted from the eight RCTs. There was no significant difference in the heterogeneity for TRs (*P*>0.05), and therefore FEM was used. The analysis showed a significant increase in grade 3–4 neutropenia (OR 1.90, 95% CI: 1.54–2.34, *P*<0.01), grade 3–4 thrombocytopenia (OR 1.62, 95% CI: 1.20–2.18, *P*<0.01) and grade 3–4 diarrhea (OR 2.04, 95% CI: 1.28–3.26, *P*<0.01), but significant increase in grade 3–4 anemia, nausea and vomiting were not observed when the GEM+5-FU/CAP/S-1 group was compared to the GEM alone group (Table 2, Figure 7). The dropout rates due to toxicity were 0–7.1% in the GEM alone group and 0.6–8.9% in the GEM+5-FU/CAP/S-1 group. However, there were no significant differences in the dropout rates between the two groups.

**Table 2:** Comparison of Grade 3–4 toxicity rates between GEM combination therapy and GEM alone therapy.

Grade 3–4 TRs	No of Grade 3–4 TRs/Total patients (%)		OR	95%CI	Significance test	
	GEM combination	GEM alone			*Z*	*P*
Neutropenia	350/979 (35.8)	238/984 (24.2)	1.90	1.54–2.34	6.03	<0.01
Thrombocytopenia	122/1021 (11.9)	80/1035 (7.7)	1.62	1.20–2.18	3.19	<0.01
Anemia	98/1020 (9.6)	91/1003 (9.1)	1.11	0.82–1.50	0.66	0.51
Diarrhea	53/979 (5.4)	26/984 (2.6)	2.04	1.28–3.26	2.99	<0.01
Nausea	57/986 (5.8)	42/994 (4.2)	1.37	0.91–2.06	1.53	0.13
Vomiting	46/882 (5.2)	39/883 (4.4)	1.18	0.76–1.83	0.76	0.45

GEM, gemcitabine; TRs, toxicity rates; OR, odds ratio; CI, confidence interval.

## Discussion

GEM is a therapy cornerstone for patients with LA/MPC. However, LA/MPC patients receiving GEM therapy have a MST of only 5.65 months [3]. In order to improve the survival benefit for LA/MPC patients, many RCTs evaluated the efficacy of GEM combined with 5-FU/CAP/S-1. In this study, we compared the efficacy and safety profile of GEM combined with 5-FU/CAP/S-1 versus GEM alone in LA/MPC patients.

Berlin's phase III study reported that the median OS was 6.7 months for GEM combined with 5-FU and 5.4 months for GEM alone (*P* = 0.09) [21]. Di Costanzo's phase II study reported that treatment with GEM combined with 5-FU obtained a median OS of 31 weeks and 30 weeks in GEM alone. But our subgroup meta-analysis obtained a meaningful conclusion that GEM combined with 5-FU significantly improved the OS and ORR compared with GEM alone [23], [29]. This conclusion supports that the addition of 5-FU to GEM maybe replace GEM in the management of LA/MPC patients.

CAP, an oral tumor-selective fluoropyrimidine, has been verified as efficacious as continuous-infusion 5-FU [30]. Both single-arm studies about GEM combined with CAP reported that the median OS were 8.7 and 10.0 months respectively in LA/MPC patients [31], [32]. Phase II and III comparison studies confirmed that the combination therapy of GEM and CAP resulted in an improved OS compared to GEM monotherapy (9.5 vs 8.2 months, 8.4 vs 7.2 months, 7.1 vs 6.2 months, respectively) in LA/MPC patients [22], [24], [25]. Moreover, Herrmann's analysis in patients with good KPS (90 to 100) showed a significant prolongation of median OS in the GEM-CAP arm compared to the GEM arm (10.1 vs 7.4 months, *P* = 0.014) [24]. In our subgroup meta-analysis, there was a significant improvement in OS (HR 0.87, *P* = 0.03) and a significant increase in ORR (OR 0.66, *P* = 0.03), but there was no significant difference in one-year survival rates between the two groups. This indicates that GEM combined with CAP maybe considered as an alternative to GEM alone, and further stratification studies are required.

S-1 is an oral 5-FU derivative with high efficiency and low toxicity. A single-arm phase II study reported that the MST was 12.5 months and one-year survival rate was 54% in LA/MPC patients receiving GEM combined with S-1 therapy [16]. Phase II and III comparison studies reported that GEM combined with S-1 did not significantly improve OS compared to GEM alone (13.5 vs 8.8 months, *P* = 0.102; 10.1 vs 8.8 months, *P* = 0.15; respectively) [26], [28]. However, in Ozaka's phase II study, the OS of patients in the GEM combined with S-1 group was significantly longer than that in the GEM alone group (13.7 vs 8.0 months, *P* = 0.035) [27]. However, two of the three studies mentioned above are open-label studies, which may result in potential bias to the conclusion. Our subgroup meta-analysis revealed that there was a significant improvement in OS and a significant increase in both one-year survival rate and ORR when the GEM combined with S-1 group was compared to the GEM group. Given these promising and surprising results, the combination of GEM and S-1 may become a valuable and acceptable alternative treatment for LA/MPC patients.

GEM combined with fluorouracil drugs brings significant clinical benefits to LA/MPC patients. Whether combination therapy leads to more side effects is also a concern for the clinican. Although this systematic assessment demonstrated a significant increase of grade 3–4 neutropenia, thrombocytopenia and diarrhea in the GEM combination group, these TRs were generally tolerable and reversible. 3 RCT (2 GEM+CAP versus GEM trials; 1 GEM+S-1 versus GEM trial) reported that the addition of CAP/S-1 to GEM did not compromise patients' quality of life or quality-adjusted life-years [22], [25], [28].

GEM-based combination therapy improved the survival benefit in LA/MPC patients. Non-GEM-based combination schemes, for example the combination of oxaliplatin, irinotecan, fluorouracil and leucovorin (FOLFIRINOX), also significantly improved OS and PFS compared to GEM alone [33]. There have been some positive results confirmed by phase III trials, and our study does not suggest that GEM combined with fluorouracil drugs surpasses other treatment schemes in certain patients [7], [33]. Rigorous phase III clinical trials are needed to further explore the potential benefits of GEM combined with fluorouracil drugs in LA/MPC patients.

This study revealed a significant improvement in OS and a significant increase in ORR when GEM combined with 5-FU/CAP/S-1 or 5-FU or CAP or S-1 were compared to GEM alone in LA/MPC patients. There was a significant increase in the one-year survival rate when GEM combined with 5-FU/CAP/S-1 or S-1 was compared to GEM alone. Grade 3–4 neutropenia, thrombocytopenia and diarrhea were significantly increased in GEM combined with 5-FU/CAP/S-1 group. The combination of GEM and fluorouracil drugs may be considered as a valuable and acceptable alternative treatment for medically fit patients with LA/MPC.

## Supporting Information

Checklist S1 PRISMA Checklist.
(DOC)

## References

[1] Goulart BH, Clark JW, Lauwers GY, Ryan DP, Grenon N, et al (2009) Long term survivors with metastatic pancreatic adenocarcinoma treated with gemcitabine: a retrospective analysis. J Hematol Oncol 2: 13. 19291303 10.1186/1756-8722-2-13 PMC2663565

[2] Bria E, Milella M, Gelibter A, Cuppone F, Pino MS, et al (2007) Gemcitabine-based combinations for inoperable pancreatic cancer: have we made real progress? A meta-analysis of 20 phase 3 trials. Cancer 110: 525–533. 17577216 10.1002/cncr.22809

[3] Burris HR, Moore MJ, Andersen J, Green MR, Rothenberg ML, et al (1997) Improvements in survival and clinical benefit with gemcitabine as first-line therapy for patients with advanced pancreas cancer: a randomized trial. J Clin Oncol 15: 2403–2413. 9196156 10.1200/JCO.1997.15.6.2403

[4] Choi JH, Oh SY, Kwon HC, Kim JH, Lee JH, et al (2008) Gemcitabine versus gemcitabine combined with cisplatin treatment locally advanced or metastatic pancreatic cancer: a retrospective analysis. Cancer Res Treat 40: 22–26. 19688061 10.4143/crt.2008.40.1.22 PMC2699081

[5] Xie DR, Yang Q, Chen DL, Jiang ZM, Bi ZF, et al (2010) Gemcitabine-based cytotoxic doublets chemotherapy for advanced pancreatic cancer: updated subgroup meta-analyses of overall survival. Jpn J Clin Oncol 40: 432–441. 20147334 10.1093/jjco/hyp198

[6] Moore MJ, Goldstein D, Hamm J, Figer A, Hecht JR, et al (2007) Erlotinib plus gemcitabine compared with gemcitabine alone in patients with advanced pancreatic cancer: a phase III trial of the National Cancer Institute of Canada Clinical Trials Group. J Clin Oncol 25: 1960–1966. 17452677 10.1200/JCO.2006.07.9525

[7] Von Hoff DD, Ervin T, Arena FP, Chiorean EG, Infante J, et al (2013) Increased survival in pancreatic cancer with nab-paclitaxel plus gemcitabine. N Engl J Med 369: 1691–1703. 24131140 10.1056/NEJMoa1304369 PMC4631139

[8] Ciliberto D, Botta C, Correale P, Rossi M, Caraglia M, et al (2013) Role of gemcitabine-based combination therapy in the management of advanced pancreatic cancer: a meta-analysis of randomised trials. Eur J Cancer 49: 593–603. 22989511 10.1016/j.ejca.2012.08.019

[9] Hu J, Zhao G, Wang HX, Tang L, Xu YC, et al (2011) A meta-analysis of gemcitabine containing chemotherapy for locally advanced and metastatic pancreatic adenocarcinoma. J Hematol Oncol 4: 11. 21439076 10.1186/1756-8722-4-11 PMC3079694

[10] Sun C, Ansari D, Andersson R, Wu DQ (2012) Does gemcitabine-based combination therapy improve the prognosis of unresectable pancreatic cancer? World J Gastroenterol 18: 4944–4958. 23002368 10.3748/wjg.v18.i35.4944 PMC3447278

[11] Maisey N, Chau I, Cunningham D, Norman A, Seymour M, et al (2002) Multicenter randomized phase III trial comparing protracted venous infusion (PVI) fluorouracil (5-FU) with PVI 5-FU plus mitomycin in inoperable pancreatic cancer. J Clin Oncol 20: 3130–3136. 12118027 10.1200/JCO.2002.09.029

[12] Chau I, Cunningham D, Russell C, Norman AR, Kurzawinski T, et al (2006) Gastrazole (JB95008), a novel CCK2/gastrin receptor antagonist, in the treatment of advanced pancreatic cancer: results from two randomised controlled trials. Br J Cancer 94: 1107–1115. 16622436 10.1038/sj.bjc.6603058 PMC2361246

[13] Cartwright TH, Cohn A, Varkey JA, Chen YM, Szatrowski TP, et al (2002) Phase II study of oral capecitabine in patients with advanced or metastatic pancreatic cancer. J Clin Oncol 20: 160–164. 11773165 10.1200/JCO.2002.20.1.160

[14] Saif MW, Syrigos KN, Katirtzoglou NA (2009) S-1: a promising new oral fluoropyrimidine derivative. Expert Opin Investig Drugs 18: 335–348. 10.1517/13543780902729412 19243284

[15] Shirasaka T (2009) Development history and concept of an oral anticancer agent S-1 (TS-1): its clinical usefulness and future vistas. Jpn J Clin Oncol 39: 2–15. 19052037 10.1093/jjco/hyn127 PMC2639406

[16] Nakamura K, Yamaguchi T, Ishihara T, Sudo K, Kato H, et al (2006) Phase II trial of oral S-1 combined with gemcitabine in metastatic pancreatic cancer. Br J Cancer 94: 1575–1579. 16721372 10.1038/sj.bjc.6603168 PMC2361295

[17] Moher D, Liberati A, Tetzlaff J, Altman DG (2009) Preferred reporting items for systematic reviews and meta-analyses: the PRISMA statement. BMJ 339: b2535. 19622551 10.1136/bmj.b2535 PMC2714657

[18] Higgins JP, Altman DG, Gotzsche PC, Juni P, Moher D, et al (2011) The Cochrane Collaboration's tool for assessing risk of bias in randomised trials. BMJ 343: d5928. 22008217 10.1136/bmj.d5928 PMC3196245

[19] Parmar MK, Torri V, Stewart L (1998) Extracting summary statistics to perform meta-analyses of the published literature for survival endpoints. Stat Med 17: 2815–2834. 9921604 10.1002/(sici)1097-0258(19981230)17:24<2815::aid-sim110>3.0.co;2-8

[20] Tierney JF, Stewart LA, Ghersi D, Burdett S, Sydes MR (2007) Practical methods for incorporating summary time-to-event data into meta-analysis. Trials 8: 16. 17555582 10.1186/1745-6215-8-16 PMC1920534

[21] Berlin JD, Catalano P, Thomas JP, Kugler JW, Haller DG, et al (2002) Phase III study of gemcitabine in combination with fluorouracil versus gemcitabine alone in patients with advanced pancreatic carcinoma: Eastern Cooperative Oncology Group Trial E2297. J Clin Oncol 20: 3270–3275. 12149301 10.1200/JCO.2002.11.149

[22] Scheithauer W, Schull B, Ulrich-Pur H, Schmid K, Raderer M, et al (2003) Biweekly high-dose gemcitabine alone or in combination with capecitabine in patients with metastatic pancreatic adenocarcinoma: a randomized phase II trial. Ann Oncol 14: 97–104. 12488300 10.1093/annonc/mdg029

[23] Di Costanzo F, Carlini P, Doni L, Massidda B, Mattioli R, et al (2005) Gemcitabine with or without continuous infusion 5-FU in advanced pancreatic cancer: a randomised phase II trial of the Italian oncology group for clinical research (GOIRC). Br J Cancer 93: 185–189. 15986036 10.1038/sj.bjc.6602640 PMC2361554

[24] Herrmann R, Bodoky G, Ruhstaller T, Glimelius B, Bajetta E, et al (2007) Gemcitabine plus capecitabine compared with gemcitabine alone in advanced pancreatic cancer: a randomized, multicenter, phase III trial of the Swiss Group for Clinical Cancer Research and the Central European Cooperative Oncology Group. J Clin Oncol 25: 2212–2217. 17538165 10.1200/JCO.2006.09.0886

[25] Cunningham D, Chau I, Stocken DD, Valle JW, Smith D, et al (2009) Phase III randomized comparison of gemcitabine versus gemcitabine plus capecitabine in patients with advanced pancreatic cancer. J Clin Oncol 27: 5513–5518. 19858379 10.1200/JCO.2009.24.2446

[26] Nakai Y, Isayama H, Sasaki T, Sasahira N, Tsujino T, et al (2012) A multicentre randomised phase II trial of gemcitabine alone vs gemcitabine and S-1 combination therapy in advanced pancreatic cancer: GEMSAP study. Br J Cancer 106: 1934–1939. 22555398 10.1038/bjc.2012.183 PMC3388559

[27] Ozaka M, Matsumura Y, Ishii H, Omuro Y, Itoi T, et al (2012) Randomized phase II study of gemcitabine and S-1 combination versus gemcitabine alone in the treatment of unresectable advanced pancreatic cancer (Japan Clinical Cancer Research Organization PC-01 study). Cancer Chemother Pharmacol 69: 1197–1204. 22249272 10.1007/s00280-012-1822-1

[28] Ueno H, Ioka T, Ikeda M, Ohkawa S, Yanagimoto H, et al (2013) Randomized phase III study of gemcitabine plus S-1, S-1 alone, or gemcitabine alone in patients with locally advanced and metastatic pancreatic cancer in Japan and Taiwan: GEST study. J Clin Oncol 31: 1640–1648. 23547081 10.1200/JCO.2012.43.3680

[29] Xie DR, Liang HL, Wang Y, Guo SS, Yang Q (2006) Meta-analysis on inoperable pancreatic cancer: a comparison between gemcitabine-based combination therapy and gemcitabine alone. World J Gastroenterol 12: 6973–6981. 17109519 10.3748/wjg.v12.i43.6973 PMC4087341

[30] Ling W, Fan J, Ma Y, Ma Y, Wang H (2011) Capecitabine-based chemotherapy for metastatic colorectal cancer. J Cancer Res Clin Oncol 137: 927–938. 20936301 10.1007/s00432-010-0954-0 PMC11828214

[31] Hubner RA, Worsnop F, Cunningham D, Chau I (2013) Gemcitabine plus capecitabine in unselected patients with advanced pancreatic cancer. Pancreas 42: 511–515. 23462324 10.1097/MPA.0b013e31826c6aee

[32] Choi JG, Seo JH, Oh SC, Choi CW, Kim JS (2012) A Phase II Trial of Gemcitabine plus Capecitabine for Patients with Advanced Pancreatic Cancer. Cancer Res Treat 44: 127–132. 22802751 10.4143/crt.2012.44.2.127 PMC3394862

[33] Gourgou-Bourgade S, Bascoul-Mollevi C, Desseigne F, Ychou M, Bouche O, et al (2013) Impact of FOLFIRINOX compared with gemcitabine on quality of life in patients with metastatic pancreatic cancer: results from the PRODIGE 4/ACCORD 11 randomized trial. J Clin Oncol 31: 23–29. 23213101 10.1200/JCO.2012.44.4869

